# Beyond Menstrual Dysfunction: Does Altered Endocrine Function Caused by Problematic Low Energy Availability Impair Health and Sports Performance in Female Athletes?

**DOI:** 10.1007/s40279-024-02065-6

**Published:** 2024-07-12

**Authors:** Johanna K. Ihalainen, Ritva S. Mikkonen, Kathryn E. Ackerman, Ida A. Heikura, Katja Mjøsund, Maarit Valtonen, Anthony C. Hackney

**Affiliations:** 1 Biology of Physical Activity, Faculty of Sport and Health Sciences, University of Jyväskylä, PO Box 35, 40014 Jyväskylä, Finland; 2 Sports Technology Unit, Faculty of Sport and Health Sciences, University of Jyväskylä, Vuokatti, Finland; 3 Wu Tsai Female Athlete Program, Division of Sports Medicine, Boston Children’s Hospital, Boston, MA USA; 4 Neuroendocrine Unit, Massachusetts General Hospital and Harvard Medical School, Boston, MA USA; 5 Canadian Sport Institute-Pacific, Victoria, BC Canada; 6 Exercise Science, Physical and Health Education, University of Victoria, Victoria, BC Canada; 7 Paavo Nurmi Centre and Unit for Health and Physical Activity, University of Turku, Turku, Finland; 8 National Olympic Training Centre Helsinki, Helsinki, Finland; 9 Finnish Institute of High Performance Sport KIHU, Jyväskylä, Finland; 10 Department of Exercise and Sport Science, University of North Carolina at Chapel Hill, Chapel Hill, NC USA; 11 Department of Nutrition, University of North Carolina at Chapel Hill, Chapel Hill, NC USA

## Abstract

Low energy availability, particularly when problematic (i.e., prolonged and/or severe), has numerous negative consequences for health and sports performance as characterized in relative energy deficiency in sport. These consequences may be driven by disturbances in endocrine function, although scientific evidence clearly linking endocrine dysfunction to decreased sports performance and blunted or diminished training adaptations is limited. We describe how low energy availability-induced changes in sex hormones manifest as menstrual dysfunction and accompanying hormonal dysfunction in other endocrine axes that lead to adverse health outcomes, including negative bone health, impaired metabolic activity, undesired outcomes for body composition, altered immune response, problematic cardiovascular outcomes, iron deficiency, as well as impaired endurance performance and force production, all of which ultimately may influence athlete health and performance. Where identifiable menstrual dysfunction indicates hypothalamic-pituitary-ovarian axis dysfunction, concomitant disturbances in other hormonal axes and their impact on the athlete’s health and sports performance must be recognized as well. Given that the margin between podium positions and “losing” in competitive sports can be very small, several important questions regarding low energy availability, endocrinology, and the mechanisms behind impaired training adaptations and sports performance have yet to be explored.

## Key Points

**Table:** 

There is insufficient scientific evidence in the sports science literature to directly link endocrine dysfunction (e.g., menstrual dysfunction) to decreased performance and blunted or decreased training adaptations. We can, however, derive the possible mechanistic links between low energy availability-induced hormonal dysfunction and negative health and sports performance outcomes in female athletes from established physiology.
Monitoring/tracking menstrual bleeding, ovulation (luteinizing hormone surge), and/or peak progesterone during the luteal phase may help to identify menstrual dysfunction associated with low energy availability (e.g., anovulation, luteal phase defect) before more severe menstrual dysfunction (amenorrhea) or marked health or performance decrements occur.
The endocrine consequences of low energy availability may negatively impact optimal training, recovery, and performance before or after menstrual dysfunction is evident. Concomitant disturbances in other hormonal axes and their impact on an athlete’s health and sports performance must be recognized.

## Introduction

Low energy availability (LEA) is a relatively common challenge for physically active and athletic populations [1]. Low energy availability can be problematic and can lead to numerous health and sports performance consequences described in relative energy deficiency in sport (REDs) [2–4]. Low energy availability refers to a mismatch between dietary energy intake to cover the energy cost of exercise, resulting in suboptimal energy for other physiological functions in the body, including the maintenance of optimal health and supporting adaptations to training [5]. Low energy availability can be adaptable (i.e., short term and accompanied by benign or even beneficial effects on health and performance), or problematic (i.e., prolonged and/or severe and accompanied by negative consequences for health and performance) [4]. An energy availability (EA) threshold of ~ 30 kcal kg^−1^ fat-free mass (FFM) day^−1^, below which disruptions to several hormonal secretory patterns were noted in as few as 4–5 days [6] has been identified in untrained adult women. Presently, a threshold of ~ 45 kcal kg^−1^ FFM day^−1^ is suggested for athletes to maintain body mass and support bodily function [7]. Although it is understood that an absolute universal threshold for EA does not exist [7], thresholds can be used to inform both research and practice.

The most studied aspect of REDs to date has been the female athlete triad (Triad) or the interrelationship between problematic LEA, menstrual dysfunction, and poor bone health (low bone mineral density [BMD] and increased risk of bone stress injuries) [8–13]. While early research suggested that the hypothalamic-pituitary-ovarian (HPO) axis was primarily responsible for bone decrements, it has become clear that the whole endocrine system, with its numerous feedback loops and various points of physiological interplay, influences athlete health, and ultimately, athlete performance, including the outcomes outlined in REDs [2–4]. Although the influences of short-term, medium-term, and long-term LEA on performance have been described in male and female individuals [14], and menstrual dysfunction as a surrogate marker of problematic LEA in female individuals has been linked to performance decrements in REDs [3, 14] (see Table 1), there are only a limited number of studies that actually assess sports performance, or performance changes related to hormonal profiles associated with menstrual dysfunction as summarized in Table 1. Three of these studies are longitudinal [15–17], two are cross-sectional [18, 19], and two are case studies [20, 21]. Three of these studies relied on self-reported menstrual status alone [17, 20, 21] while four studies used urinary or blood samples (or their combination) to assess endocrine (menstrual) function [15, 16, 18, 19]. Oligomenorrhea and amenorrhea were most commonly compared to natural/eumenorrheic menstrual cycles while other types of menstrual dysfunction were excluded [19] or not considered/reported. Five studies assessed endurance performance using season best or laboratory testing [15–19], while three studies assessed measures related to strength or power [20, 21], and one study used a published points system [17]. Current research in Table 1 indicates that menstrual dysfunction (e.g., ovarian suppression such as amenorrhea) generally decreases or blunts athletic performance and development whereas natural/eumenorrheic menstrual cycles tend to support performance and athletic development. Regrettably, the relatively limited scope (performance measures) and depth (assessment of mechanisms) of this research hinders our ability to extrapolate results to larger populations and to draw robust conclusions regarding the links between hormone profiles and performance. As such, a gap exists in our understanding regarding the effects of the spectrum and progression of the hormonal profiles characteristic of menstrual dysfunction on sports performance. While most LEA and REDs literature focuses on the components of the Triad, athlete health and performance comprise several other factors, including cardiovascular and ventilatory responses, substrate metabolism, neuromuscular function, nervous system activity, thermoregulation, and psychological factors, all of which are highly pertinent in a sports setting.

**Table 1:** Summary of original research linking menstrual dysfunction (either as indicators of, or secondary to, low energy availability) to sports performance

Reference	Study population Training background, participant classification (i.e., tier) [27] sample size (*n*), menstrual status, age (mean ± standard deviation or range), study design	Menstrual status classification^a^ Eumenorrheic, oligomenorrheic, amenorrheic, use of HC at the time of the study	Menstrual status assessment method Self-report, hormonal confirmation?	Hormonal results (mean ± standard deviation)	Performance assessment method and reported changes in performance
De Souza et al. [18]	Tier 2–3 runners (*n* = 16) 8 EUM (age: 29.0 ± 4.2 y) 8 AME (age: 24.5 ± 5.7 y) Cross-sectional study	AME: absence of menstruation for 3 or more consecutive months EUM: consistent recurrence of menstruation at intervals of 23–33 days HC use: no	Urinary LH and P4 assays (pre, during, and post from day 5 following the onset of menses) Ovulation confirmed Blood sample: pre-exercise E2 and P4 on early follicular and midluteal phases	EUM *Follicular phase* E2: 153.4 ± 71.3 pmol L^−1^ P4: 1.5 ± 0.8 nmol L^−1^ *Luteal phase* E2: 549.2 ± 258.2 pmol L^−1^ P4: 40.7 ± 13.9 nmol L^−1^ AME E2: 97.1 ± 68.3 pmol L^−1^ P4: 0.9 ± 0.4 nmol L^−1^	**Maximal and submaximal (40 min at 80% *V*O**_**2max**_**) treadmill run** Neither menstrual phase (follicular vs luteal) nor menstrual status (EUM vs AME) altered or limited exercise performance
Schaal et al. [15]	Tier 2 runners (*n* = 16) EUM 9 Well-adapted (age: 29.4 ± 1.6 y) 7 Non-functionally over-reaching (age: 27.7 ± 2.3 y) Longitudinal observational study	Abnormal menstrual cycle: cycle > 35 days no LH surge detected, with no mid-cycle estradiol rise luteal phase lasting < 10 days EUM: not abnormal menstrual cycle HC use: not reported	Urinary LH test (daily, starting on day 5 of the cycle until confirmation of LH surge was obtained) Ovulation confirmed Daily salivary samples: E2	*Well-adapted runners:* No change *Non-functionally over-reaching runners*: Decrease in EA was accompanied by a significant reduction in E2 at the mid-cycle point (27%) and in the first 5 days of the luteal phase (47%) 17% reduction in leptin level	**A standardized graded treadmill running test** 7/16 athletes were diagnosed as “overreaching” and after a 2-week recovery period still displayed impaired running performance and were further categorized as non-functionally overreached. No group differences in any of the training characteristics during the 3 phases
Tornberg et al. [19]	Tier 4 endurance athletes (*n* = 30) 16 EUM (age: 27.6 ± 5.6 y) 14 FHA (age: 26.1 ± 5.6 y) Cross-sectional study	EUM: menstrual cycles of 28 ± 7 days and normal sex hormones FHA: absence of menstruation for ≥ 3 consecutive months HC use: no	Urinary LH test Transvaginal ultrasound examination to screen for PCOS Self-reported cycle status. Menstrual bleeding calendar with monthly follow-up for ≥ 3 months) *No other menstrual dysfunctions other than FHA in cohort*	FHA athletes compared to EUM: Lower E2 (0.12 ± 0.03 vs 0.17 ± 0.09 nmol L^−1^, *p* < 0.05) Lower T3 (1.4 ± 0.2 vs 1.7 ± 0.3 nmol L^−1^, *p* < 0.01) Higher cortisol levels (564 ± 111 vs 400 ± 140 nmol L^−1^, *p* < 0.05) Lower blood glucose (3.8 ± 0.3 vs 4.4 ± 0.3 mmol L^−1^, *p* < 0.001)	**Incremental bicycle test, reaction time, isokinetic knee muscular strength, and endurance** FHA athletes had higher work efficiency and lower body weight than EUM athletes EUM athletes had greater knee muscular strength (11%) and knee muscular endurance (20%), and 7% shorter reaction time compared with FHA athletes Knee muscular strength and knee muscular endurance positively associated with fat-free mass of legs and T3, negatively with cortisol Faster reaction time was associated with higher blood glucose, T3, and E2 levels and lower cortisol
Vanheest et al. [16]	Tier 3 swimmers (*n* = 10) 5 Ovarian suppressed (age: 17 ± 1.7 y) 5 Cyclic menstrual function (age: 16.2 ± 1.8 y) Longitudinal observational study	Ovarian suppressed*:* P4 levels < 15.9 nmol L^−1^ on weeks 0 and 2, absence of cyclic increases in E2 Cyclic menstrual function: P4 serum levels during the luteal phase > 15.9 nmol L^−1^ and E2 > 25 pg:mL^−1^ duringthe 4-week period HC use: not reported	Blood sample: E2 and P4 at weeks 0 and 2 Self-reported menstrual status from daily logs (12 weeks)	Ovarian suppressed athletes: suppressed pattern in both ovarian steroids compared with the cyclic menstrual function group, differences in E2 at weeks 2, 6, and 10 T3 was 19% lower in ovarian suppressed group compared with the cyclic menstrual function group	**Maximal time trial performance (400-m swim)** Each swimmer with cyclic menstrual function improved 400-m performance baseline vs week 12, 8.2% improvement in the group Each ovarian-suppressed swimmer showed a decline in 400-m velocity on week 12 vs baseline, a 9.8% decline in the group. Ovarian suppressed time trial performance at week 12 declined by 10% compared with week 4 Significant relationships between week 12 and 400-m time trial velocity in P4, E2, IGF-1, T3, energy intake, and EA
Areta [20]	Tier 4–5 cyclist (*n* = 1) Age: 23 (5-y follow-up) AME 4 y OLI 4 y EUM Longitudinal observational case study	AME: no menstrual bleeding between 2013 and 2015 OLI: irregular cycles every 2–8 months with light bleeding between 2015 and 2018 EUM: resumption of regular menses, late 2018 HC use: no	Self-reported	Regular menses resumed after > 4 years of menstrual dysfunction and 5–6 months after body mass gain while maintaining a high training load	**Mean maximal power (crank-based cycling power meters)** The highest relative mean maximal powers (5 min to 1 h) were achieved during the period of menstrual dysfunction, whereas the best absolute mean maximal power across a range of durations were achieved after body mass gain and while the athlete was EUM
Ihalainen et al. [17]	Tier 3 runners (*n* = 21) 8 AME athletes 5 EUM athletes 8 EUM controls Age = 16–22 y Longitudinal observational study	AME: absence of menstruation for ≥ 3 consecutive months HC use: not reported	Self-reported	–	**800–10,000 m season best and corresponding International Amateur Athletic Federation points** AME athletes had significantly lower running volume and more injury days than EUM athletes Only EUM runners increased their performance
Tinsley et al. [21]	Tier 2–3 physique athlete (*n* = 1) Age: 27 y Longitudinal observational case study	No regularly occurring menstrual cycle, one menstrual period during the 8 months of assessments (3 months before the first competition). Ovulation resumed approximately 3 months after the second competition HC use: not reported	Self-reported	–	**Isometric and isokinetic squat** Concentric and eccentric peak forces declined up to 19% before the first competition, experienced perturbations in the inter-competition and recovery periods and remained 5–8% below baseline after 8 months Rate of force development decreased 57% before the first competition, was partially recovered, but remained 39% lower than baseline at study termination

*AME* amenorrheic, *E2* estradiol, *EA* energy availability, *EUM* eumenorrheic, *FHA* functional hypothalamic amenorrhea, *HC* hormonal contraception, *IGF-1* insulin-like growth factor 1, *LH* luteinizing hormone, *P4* progesterone, *PCOS* polycystic ovary syndrome, *T3* triiodothyronine, *VO*_*2max*_ maximal oxygen uptake, *y* year/years

^a^Participants in this table are referred to as “eumenorrheic” or “amenorrheic” as per the language used in the original publications, even when the current preferred methods for the classification of menstrual status were not used [28]. Most “eumenorrheic” participants here would be classified as “naturally menstruating” based on current recommendations [28]. In this table, “amenorrheic” is used when authors did not indicate functional hypothalamic amenorrhea

^b^Participant classification (i.e., tiers) is as per McKay et al. [27]; 0, sedentary; 1, recreationally active; 2, trained; 3, national level/highly trained; 4, international level/elite; 5, world class

The aim of this narrative review is to describe the link between LEA-induced hormonal dysfunction and the various health and sports performance outcomes in female athletes. We focus on describing key evidence-based hormonal pathways responsible for the normal physiological function necessary for sports performance. The review is divided into two parts. Part A: Beyond Menstrual Dysfunction (Sect. 2) illustrates how menstrual dysfunctions (particularly functional hypothalamic amenorrhea [FHA]), per se, are not in themselves the problem for sports performance, but rather that the altered endogenous hormone profiles, characterized by sex hormone deficiencies, contribute to dysfunction in mechanisms that affect both health and ultimately also sports performance. Part B: Beyond Menstrual Dysfunction and Sex Hormones (Sect. 3) describes how the altered endogenous sex hormone profiles associated with menstrual dysfunction are not the only hormonal challenge that arises from problematic LEA and how concurrent dysfunction in other hormonal axes contributes to impairment in mechanisms that affect athlete health and sports performance. Our description of the endocrine consequences of LEA in female athletes is relatively brief, as there are already several excellent reviews on this topic [22–26].

## Part A: Beyond Menstrual Dysfunction

The HPO axis controls female reproduction via the menstrual cycle [29]. Ideally, gonadotropin-releasing hormone (GnRH) from the hypothalamus stimulates the release of follicle-stimulating hormone (FSH) and luteinizing hormone (LH) from the anterior pituitary, stimulating follicular growth and ovulation, in addition to activating the ovaries to produce estradiol (E2) and inhibin. After ovulation, the follicle remnant becomes the corpus luteum, which is responsible for the production of progesterone (P4). While LH and FSH are important for production of the E2, LH also stimulates thecal cells in the ovaries to secrete testosterone and FSH stimulates granulosa cells in the ovarian follicles to produce aromatase, which then converts thecal cell-produced testosterone into E2. In healthy pre-menopausal women, E2 is the major circulating estrogen, playing a fundamental role in reproduction via the menstrual cycle, as well as in the physiology of the cardiovascular, skeletal, metabolic, and central nervous systems [30–32]. Similarly, P4 has several non-reproductive functions related to the cardiovascular system, central nervous system, and bone [33]. For example, P4 influences thermoregulation, ventilation, and metabolism while also having antiestrogenic and androgenic functions [34].

Several other hormones/systems contribute to the regulation of the HPO axis. For example, kisspeptins act via the kisspeptin receptor to stimulate the pulsatile release of GnRH [29]. The kisspeptin system appears to respond to both metabolic status and EA [35]. Kisspeptin activity is decreased by LEA, which, in turn, increases orexigenic factors (ghrelin) and decreases anorexigenic factors (leptin) [36]. This decrease in kisspeptin leads to a downregulation of GnRH thus influencing downstream cascades that affect appetite and feeding behavior [37]. The activin-follistatin-inhibin axis also contributes to regulation of the HPO axis, with activin increasing the synthesis/secretion of FSH and inhibin downregulating it. Inhibin secretion is reduced by GnRH and increased by insulin-like growth factor-1 (IGF-1). Similarly, glucocorticoids, such as cortisol, suppress pituitary gonadotroph responsiveness to hypothalamic input, which may also result in disruptions to the HPO axis [38].

### LEA and Menstrual Dysfunction

**Fig. 1:**
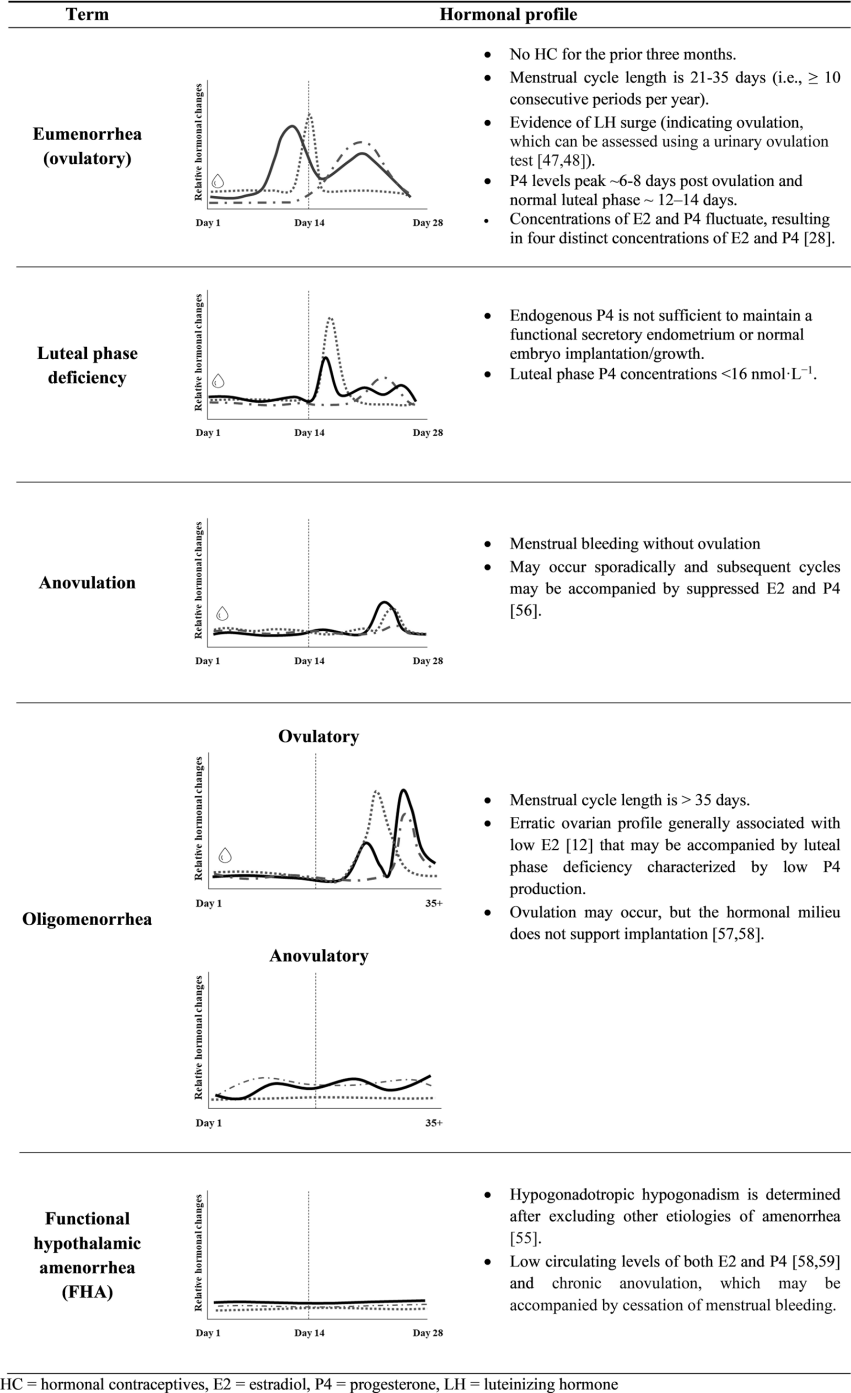
Terminology of menstrual function and dysfunction including representative hormonal profiles. Of note, hormonal profiles of hormonal contraceptive (HC) users (including combined HCs and progestin only) may be different. The solid line represents estradiol (E2), the dashed line represents progesterone (P4), the dotted line represents luteinizing hormone (LH), and the drop symbol represents menstrual bleeding. Modified from Allaway et al. [45] with permission

The HPO axis requires sufficient energy and nutrients to maintain normal menstrual function [39] or eumenorrhea (i.e., “normal” ovulatory cycles of approximately 21–35 days). Both LEA and stress (emotional and/or physical) may lead to the downregulation of the HPO axis [22, 40] both in the short term [6] and particularly when LEA is problematic or severe (< 10 kcal kg^−1^ FFM day^−1^) [41]. Downregulation of the HPO axis is indicated by changes in hormonal profiles, characteristic of menstrual dysfunction, that are recognized as a hallmark of problematic LEA and range in severity from subtle luteal phase defects to anovulation, oligomenorrhea, and secondary amenorrhea (i.e., FHA) [42–45] (definitions and representative hormonal profiles of menstrual function and dysfunction in Fig. 1). A prolonged follicular phase and luteal phase deficiency characteristic of oligomenorrhea may affect fertility [46], while the occurrence of anovulatory cycles (which can be assessed using a urinary ovulation test [47, 48]) and FHA profoundly impact fertility [49]. Yet menstrual dysfunction is potentially reversible [50] if the root cause is addressed [50]. Regrettably, without regular monitoring/tracking hormones, these changes in hormonal profiles may go unnoticed until attempting pregnancy.

A dose–response relationship has been reported between the magnitude (energy deficit of − 470 to − 810 kcal day^−1^) of LEA and the incidence of menstrual dysfunction in exercising women. However, the severity of menstrual dysfunction appears unrelated to LEA magnitude [51] and there is limited evidence for a specific EA threshold below which menstrual dysfunction is induced [52]. The prevalence of the more severe menstrual dysfunction, such as FHA, is relatively high in elite runners (self-reported = 23/36 of athletes surveyed) [50] and in other endurance athletes (clinically verified = 24/40 of athletes examined) [53]. As FHA is considered a heterogeneous group of disorders that can manifest similarly [54], diagnosis should only be confirmed after other etiologies are excluded [55]. In practice, estimation of ovulation via the LH surge and confirmation of the mid-luteal peak in progesterone indicates normal hormonal function, whereas regular menstrual bleeding alone is not an indicator of eumenorrhea [28].

### Sex Hormones and Health

While the spectrum of menstrual dysfunction (oligo/amenorrhea), as a manifestation of HPO axis dysfunction, is a commonly identified outcome of LEA in women not using hormonal contraceptives (HCs), the non-reproductive actions of suppressed hormones such as E2 and P4 also have the potential to affect health, training responses and adaptations, and ultimately sports performance. Endogenous E2 affects metabolism [32], cardiovascular function [56], bone [57], and muscle [58, 59]. Likewise, endogenous P4 influences thermoregulation, ventilation, and metabolism while having antiestrogenic and androgenic functions [34]. The wide encompassing effects of E2 and P4 are beyond the scope of this review, and we will therefore focus on the effects of E2 and P4 that are most pertinent to sports performance.

#### Bone

Energy availability and E2 independently and synergistically affect volumetric BMD, bone geometry, and estimates of bone strength [57]. Overall poor bone health is also associated with other LEA-induced hormonal disruptions including decreases in androgens, insulin, IGF-1, triiodothyronine (T3), and leptin in addition to increases in fasting peptide YY (PYY), ghrelin, and cortisol [22, 60, 61]. Athletes and non-athletic women with LEA, as well as athletes with FHA, have lower BMD, impairments of bone microarchitecture, and altered markers of bone remodeling compared with those with adequate EA and eumenorrhea [62–64]. Athletes with menstrual dysfunction (oligo/amenorrhea) also have decreased bone strength estimates and higher lifetime fracture rates compared with both eumenorrheic athletes and controls [65, 66]. Women participating in leanness sports have higher rates of menstrual dysfunction, low BMD, and fracture than other sports [67, 68]. Indeed, the prevalence of bone stress injuries is higher in amenorrheic athletes than naturally menstruating athletes [68, 69], whereas even short-term manipulation of EA (15 vs 45 kcal kg^−1^ FFM day^−1^) in naturally menstruating women performing daily endurance exercise decreased bone formation and increased bone resorption marker concentrations [70, 71]. In practice, detrimental structural changes in bone resulting from low E2 and accompanying hormonal dysfunction induced by LEA may be undetected for years, but the consequences of low BMD and recurrent bone stress injuries have significant repercussions on both health and ultimately performance (via modified and missed training days). It should be highlighted that the risk for bone stress injuries related to the Triad is found to be higher in teenage athletes than for athletes in their twenties [72]. Furthermore, the accrual of lost BMD when EA is corrected (depending on the timing and duration of LEA) may be difficult, if not impossible [73, 74]. As such, avoidance of LEA and menstrual dysfunction is essential for long-term bone health.

#### Body Composition

Estrogens are important for the regulation of body weight and body composition. Estrogens influence fat distribution and are associated with lower visceral fat [75]. Endogenous E2 is an anabolic hormone associated with muscle mass and strength in female athletes [59]. Estradiol plays a role in facilitating muscle tissue sensitivity to anabolic stimuli, regulating myofibrillar protein synthesis [59] and skeletal muscle hypertrophy [58, 76]. Endogenous E2 upregulates intracellular signaling pathways that stimulate muscle protein synthesis [77] and may play a role in muscle repair and regeneration [58]. Low energy availability-induced low E2 may affect muscle quality, as E2 is known to protect muscles from damage by acting as an antioxidant or membrane stabilizer or by affecting gene regulation [58] while having antiapoptotic effects [78]. Indeed, estrogen receptors are found in several tissues and organs of the body and are known to modulate cell proliferation, differentiation, and survival. Estrogens also exhibit neuroprotective capabilities by promoting DNA repair, stimulating growth factor expression, and modulating blood flow, whereas E2-dependent signaling pathways are involved in neurogenic processes [79]. Ultimately, ineffective tissue repair and regeneration may impair training adaptations and athletes with low E2 may be more susceptible to muscle damage (i.e., extended recovery times). Generally, lean body composition and low body weight are associated with performance in endurance sports. Lower body fat is associated with better endurance performance while gains in muscle mass are generally associated with increases in performance across sports [80]. A decrease in body mass due to LEA may increase maximal aerobic capacity relative to body mass (maximum oxygen uptake in mL kg^−1^ min^−1^), even in the absence of changes in absolute aerobic capacity (maximum oxygen uptake in mL min^−1^); however, the benefits are likely to be transient when prolonged LEA and menstrual dysfunction are present. Indeed, lower body weight and fat mass in elite amenorrheic endurance athletes do not appear to result in improved aerobic capacity compared with eumenorrheic athletes [19].

#### Cardiovascular System

Systemic vascular circulation is an important component of health and performance. In a healthy blood vessel, E2 is a potent vasodilator via nitric oxide production; it also mediates inflammation and oxidative stress [81]. Short-term perturbations in E2 might influence blood flow via disturbed endothelial function and low E2 associated with menstrual dysfunction has been linked to lower blood pressure and heart rate response [56]. Perturbations in circulation may impair the transport of oxygen and energy substrates, including glucose and fatty acids, to skeletal muscle, while clearance of metabolic waste may also be affected. Physically active women with low E2 demonstrate lower heart rate and blood pressure response to an orthostatic challenge in which plasma renin, angiotensin II, and aldosterone fail to increase, resulting in a sympathetic vasoconstrictor response to compensate for blood pressure changes [56].

Importantly, LEA may cause endothelial dysfunction independently of low E2 [82, 83] and extreme LEA can lead to cardiac arrythmias [84]. The effects of P4 on the cardiovascular system have received less attention, although there is evidence that P4 lowers blood pressure, inhibits coronary hyperactivity, and has powerful vasodilatory and natriuretic effects [85]. Vascular dysfunction caused, in part, by reduced E2 may be accompanied by impaired/blunted nitric oxide production; early signs of cardiovascular dysfunction have been identified in young amenorrheic athletes including an unfavorable lipid profile: higher total cholesterol and low-density lipoprotein cholesterol [86]. Likewise, reduced endothelium-dependent vasodilation [83], increased vascular tone, lower shear rate, as well as impaired endothelial and/or vascular smooth muscle cell responsiveness to nitric oxide have been reported in female athletes with LEA-induced amenorrhea [82, 86–89]. Taken together, low E2-induced and P4-induced changes in the circulation and cardiovascular function may, in theory, influence training responses and quality, as well as subsequent adaptations and/or performance.

Despite the unfavorable lipid profile that may present in athletes with LEA, it is important to remember that cholesterol is essential for the metabolism of steroid hormones. Cholesterol is, for example, metabolized to pregnenolone, which is then further metabolized into sex steroids E2 and P4 [90]. As such, it is possible that the observed high cholesterol associated with menstrual dysfunction is a compensatory mechanism for decreased E2 and P4 in LEA or that the metabolism of cholesterol into steroid hormones is disturbed by LEA.

### Summary Part A

Menstrual dysfunction is not in itself a problem for sports performance, but the altered endogenous hormone profiles, characterized by sex hormone deficiencies, contribute to dysfunction in mechanisms that affect health, training quality, and sports performance.

## Part B: Beyond Menstrual Dysfunction and Sex Hormones

Beyond the HPO axis, several other hormonal axes are affected by LEA. Together, the hypothalamus and pituitary gland control downstream processes related to an athlete’s health and sports performance, including autonomic, endocrine, and somatic responses and adaptations. For example, the hypothalamic–pituitary–adrenal (HPA) axis regulates responses to stress and plays a critical role in energy metabolism, particularly in relation to food intake, energy storage, and energy mobilization [22]. As a catabolic and glucoregulatory hormone, downstream cortisol is secreted in response to physical stress and other challenges to body homeostasis [91]. In turn, the hypothalamic–pituitary–thyroid axis controls metabolic hormones that play a key role in regulating musculoskeletal health and function [92], while several other hormones, including leptin, ghrelin, insulin, and PYY regulate EI via appetite regulation and/or behavioral food intake. Some of these hormones have additional functions, for instance regulating gastric motility, water and electrolyte absorption, and immunological responses [93–95].

Hormones such as growth hormone (GH), IGF-1 and its binding proteins, insulin, and testosterone are important for anabolic processes and are major determinants of body composition [96]. Insulin-like growth factor-1 plays a direct role in whole-body glucose homeostasis, influences muscle hypertrophy [97], and is positively associated with muscular endurance and aerobic fitness [98]. Growth hormone modulates insulin sensitivity, glucose homeostasis, and metabolic response to calorie restriction. Importantly, the GH-IGF axis also influences immunity and inflammation [99]. Insulin acts as an anabolic/anticatabolic hormone, mitigating muscle protein breakdown [100, 101] with similar actions by GH, which primarily acts via its actions on IGF-1 [100]. More specifically, IGF-1 is involved in managing muscle protein synthesis, hypertrophy, and inhibition of muscle protein breakdown [102]. Testosterone is produced in female individuals by the ovary, adrenal glands, and peripheral tissues via conversion of androstenedione and dehydroepiandrosterone (pre-androgens synthesized by the ovaries and adrenal glands) to testosterone. Testosterone has both direct and indirect (via aromatization to E2) functions related to vasomotor tone, endothelial function, peripheral vascular resistance, cognition, and musculoskeletal health [103].

### LEA and Endocrine Dysfunction

Short-term LEA has been shown to elevate blood cortisol in a non-linear pattern in naturally menstruating women. A decrease in EA from 45 to 30 or 20 kcal kg^−1^ FFM day^−1^ was associated with a small increase in blood cortisol, whereas a more notable increase (~ 150%) was observed at an EA of 10 kcal kg^−1^ FFM day^−1^ [6]. However, significant changes in blood cortisol levels were not observed in bodybuilding fitness athletes after a 4-month fat-loss diet combined with a high training volume [104]. In elite female endurance athletes with varying levels of EA, cortisol levels were highest in women reporting menstrual dysfunction compared with their regularly menstruating counterparts [53], which is consistent with previous research [105, 106].

Laboratory-based interventions and cross-sectional investigations have reported decreases in T3, leptin, insulin, and IGF-1, as well as increased growth hormone (GH) and adiponectin due to LEA [22, 23]. Short-term investigations in healthy sedentary women have shown decreased 24-h mean levels of insulin and leptin with decreasing EA (from an adequate EA of 45 kcal kg^−1^ FFM day^−1^). In fact, when EA decreased from 45 to 30 kcal kg^−1^ FFM day^−1^, there was a 35% decrease in leptin with a further decrease (~ 70%) at an EA of 10 kcal kg^−1^ FFM day^−1^. Decreases in fasting levels of IGF-1 and T3 occurred at a threshold of ~ 20–25 kcal kg^−1^ FFM day^−1^ [6]. More recently, short-term LEA (15 kcal kg^−1^ FFM day^−1^) decreased fasting levels of insulin and leptin in eumenorrheic female individuals when compared with adequate EA [71]. Similarly, a short-term diet or exercise-induced LEA reduced fasting levels of IGF-1, leptin, and T3 [107]. Cross-sectional investigations comparing metabolic hormone profiles between amenorrheic and eumenorrheic female individuals confirm these findings, showing both lower levels of T3 [16, 19, 69] and leptin [108, 109] in athletes with menstrual dysfunction. Similarly, ghrelin levels were higher after 12 weeks of reduced EA that resulted in a minimum of 1.5 kg of weight loss, whereas no change in the anorexigenic PYY was observed [110]. Nevertheless, ghrelin and PYY have been found to be higher in amenorrheic versus eumenorrheic athletes [61, 111].

Research on the relationship between LEA/amenorrhea on androgens in female individuals has so far yielded equivocal results with reports of both decreased [105, 112] and increased [113, 114] levels of androgens. For example, lower levels of testosterone have been reported in amenorrhea and oligomenorrhea resulting from LEA and chronic energy deficit states [105]. Similarly, oligomenorrheic and amenorrheic athletes, in comparison to eumenorrheic athletes, had lower testosterone and dehydroepiandrosterone (DHEA) sulfate levels, as well as higher sex hormone binding globulin (SHBG) levels [112]. Higher levels of testosterone in dancers with menstrual dysfunction (and low daily energy and carbohydrate intake) have been reported (in those without characteristics of hyperandrogenism/polycystic ovary syndrome) [113]. Likewise, endurance athletes with oligomenorrhea or amenorrhea were reported to have higher serum levels of both free and total testosterone as well as androstenedione, which was accompanied by lower SHBG levels when compared with eumenorrheic endurance athletes and non-athletes [114]. Levels of SHBG may help explain differences in androgen availability as SHBG has a high affinity and specificity for binding sex hormones where serum levels are regulated by androgens, estrogens, thyroid hormones, as well as other metabolic factors including EA and physical activity [115]. Sex hormone binding globulin binds to E2, dihydrotestosterone, and testosterone, rendering these hormones biologically inactive. However, higher levels of testosterone in dancers [113] and endurance athletes with menstrual dysfunction [114] could be explained by HPO axis suppression of FSH release, which inhibits aromatase production, potentially resulting in low E2 and high testosterone. Higher testosterone could function as a compensatory mechanism, as testosterone is aromatized to E2, but could also be the result of elevated adrenal activity [116] or due to a decrease in adipose tissue [117]. While the precise mechanisms behind these observations are unclear, other causes of hyperandrogenism (e.g., adrenal hyperplasia, polycystic ovary syndrome) should be considered, as the current LEA and sports science literature does not consistently screen for and exclude other causes of hyperandrogenism. While the effects of LEA on androgens and androgen precursors in women are under-studied and results are inconsistent [118], LEA-induced perturbations in androgen levels in female individuals may influence, among other things, musculoskeletal health [103].

### Other Hormones and Health

A spectrum of downstream hormones are affected by LEA, leading to disturbances in normal physiological and physical function that manifest as metabolic and immunological challenges. These are addressed in the following sub-sections.

#### Metabolism and Management of Body Composition

In conditions of problematic LEA, the resting metabolic rate (RMR) is reported to decrease, thus affecting the management of body composition in athletes. Indeed, several studies suggest links between problematic LEA, suppressed metabolic hormones, and suppression of RMR. The body has several regulatory systems for mitigating weight loss [119, 120]. For example, leptin acts on the RMR indirectly by suppressing T3 and the activity of the sympathetic nervous system. In addition, decreases in the RMR due to energy restriction may be a result of suppressed catecholamine and thyroid hormone levels [121]. Indeed, reduced RMR has been linked to lower T3 and leptin levels [122, 123], while neither body mass nor FFM appears to explain differences in RMR [123, 124]. Although a difference in body mass or relative fat mass is not consistently observed between amenorrheic and eumenorrheic female individuals, the former appear to have lower levels of T3 [69] and lower RMR [53, 125].

In conditions of LEA, exercise energy expenditure (EEE) tends to decrease [124], contributing to a reduction in total daily energy expenditure, which may affect weight management. The endocrine changes resulting from LEA also appear to affect muscle efficiency and EEE. For example, a 10% loss of body mass led to a 20% increase in skeletal muscle work efficiency during a bicycle ergometer test with light workloads (10, 25, and 50 W), accounting for ~ 75% of the decline in EEE [126]. Similarly, Tornberg and colleagues [19] reported a lower RMR, as well as lower EEE during cycling, concurrent with lower levels of T3 levels in amenorrheic versus eumenorrheic female athletes.

Prolonged concomitant reductions in RMR and EEE are likely to translate into an inability, or extreme difficulty, to maintain or lose body mass, thereby challenging the management of body composition in the athlete. Hormones are also major regulators of muscle protein turnover [77, 100], which has additional implications for the management of body composition, as well as the strength and power capabilities of an athlete. Leanness/thinness may be associated with some forms of menstrual dysfunction [19, 53, 86, 109], but athletes in some sports perceive theoretical benefit from a lean body composition with lower levels of adiposity and higher levels of muscle mass [127, 128]. Lower fat percentage may not actually be beneficial, as has been reported in cross-sectional [129] and longitudinal [130] studies. Most reports characterizing body composition in amenorrheic and eumenorrheic female individuals indicate lower body mass and fat mass in the former group [19, 53, 86, 109]. Whether this is an outcome of LEA, but eventually leads to issues including overcompensation (storage of extra energy as adipose tissue) to sudden increases in EA following a prolonged and/or severe period of LEA [119], remains to be elucidated. Importantly, the hormonal changes associated with long-term LEA are not favorable for maintaining healthy body composition.

Anabolic responses to exercise may be blunted in conditions of LEA [131]. This is supported by findings of GH resistance along with higher levels of cortisol in women with anorexia nervosa [132]. Importantly, nutritional status appears to outweigh the effects of cortisol on GH levels [132]. Nevertheless, glucocorticoids directly inhibit IGF-1 induction of the molecular pathways that stimulate muscle protein synthesis while IGF-1 appears to at least partially reverse glucocorticoid-induced muscle protein breakdown [133]. Areta and colleagues reported a 27% reduction in resting muscle protein synthesis after only 5 days of EA of 30 kcal kg^−1^ FFM day^−1^ in both female and male individuals [134]. Low glycogen, a likely consequence of LEA, has been demonstrated to negatively affect cellular growth and adaptation in response to resistance exercise independently of hormonal responses [135], while exercise nutrient interactions influence cascades that affect protein regulatory systems during both exercise and recovery where energy is also needed to fuel cellular pathways [136]. Athletes experiencing short-term LEA might be less prone to muscle catabolism seen in long-term or the most severe forms of LEA, but it is expected that optimal rates of muscle protein synthesis will suffer [137], thus blunting responses and adaptations to resistance training that would otherwise result in muscle hypertrophy. Resistance exercise and amino acid ingestion are crucial to stimulate anabolism, but physiological stress, including LEA, attenuates these effects [138]. Concomitant with nutrient (amino acid and carbohydrate) deficiency, muscle protein synthesis, and muscle remodeling may also be affected by increased catabolic cortisol and decreased levels of anabolic hormones (GH, IGF-1, E2) [139]. Ultimately, impaired muscle protein synthesis and subsequent effects on lean mass may have dramatic implications for sports performance.

#### Immune Function

The consequences of LEA-induced endocrine dysfunction may predispose athletes to illness [140, 141] as well as injury [69, 142], with endocrine dysfunction affecting the time course of return to play. Indeed, illness and inflammation influence an athlete’s potential to train and compete, while also affecting recovery and healing. Sports that combine exercise training with LEA to modify weight and body composition appear to influence immune function [104, 141–146]. Importantly, LEA during recovery from illness/injury may further complicate or delay healing/immune processes whereas the nutritional component of healing is often overlooked [147]. Injury/illness alters an athlete’s nutritional requirements [148], where negative energy balance is known to impair wound healing [149] and increase muscle loss [150] due to down-regulation of muscle protein synthesis and associated intracellular signaling proteins, even during a moderate decrease in EA [134]. It is possible that LEA-induced alterations in hormones concurrent with LEA-induced nutritional deficiencies increase athletes’ susceptibility to illness/infection and injury, while predisposing them to injury cycles due to suboptimal healing. Although several mechanisms related to LEA may reduce the activation and efficacy of the immune system, the significance of a single factor, such as EA, remains unknown.

Many aspects of exercise-induced modifications in immune function may be mediated by increased levels of immunoregulatory hormones such as cortisol [151] while actions of immune cells are also known to be modulated by E2 [152]. Antiviral mechanisms may be modified in women with hormonal profiles associated with menstrual dysfunction. The literature indicates that E2 activates monocytes, macrophages, and neutrophils, which induce the production of proinflammatory cytokines [152]. Similarly, E2 and P4 have been shown to blunt the interleukin-10 response [153], which is associated with more infections in athletes [154]. Finally, E2 promotes hydration of mucous membranes, which could influence the local immune response [155]. The protective mechanism of E2 could be mediated by the increase in the production of nasal mucus that contains immunoglobulin A, an immunoglobin negatively associated with the incidence of respiratory infections in athletes [154]. In a related way, irritative urinary symptoms (including urinary tract infections) may be problematic and indicative of such events in female athletes [156].

#### Gut Health

LEA can lead to gastrointestinal distress in athletes [157]. Gut health in the context of LEA-induced hormonal dysfunction has not been extensively addressed in the literature, although gut health and function are of great importance to athletes. The gut plays an essential role in the digestion and absorption of nutrients, while also providing a barrier between the external environment and circulation (immune function). Digestion can be impaired during LEA, with symptoms such as constipation, diarrhea, and slowed gastric emptying [158]. In extreme LEA, (e.g., in patients with anorexia nervosa) gut microbiota diversity and richness are reduced, which has been suggested to be linked to compromised bone health [159]. There appears to be a bidirectional relationship between the gut microbiota and sex hormones, although research in athletic female populations is sparse [160]. Nevertheless, E2 is known to strengthen and protect the mucosal and epithelial barriers in the gastrointestinal tract while modulating both intestinal inflammation and immune response [161]. The gastrointestinal tract microbiome contributes to immune function, regulates systemic inflammation, and appears to affect higher cognitive functions [162]. In this sense, gut microbiota may regulate to some degree oxidative stress, inflammatory responses, metabolism, and energy expenditure during intense exercise [163]. While additional research is required, cross-sectional studies have reported associations between physical performance and gut microbiota status [164].

#### Iron

It has been demonstrated that LEA interacts with iron deficiency [165], where prolonged LEA, concomitant with an inadequate iron intake, can have negative effects on iron stores and eventually hemoglobin, both of which can subsequently affect sports performance [166]. Iron deficiency interacts with LEA to perturb thyroid function and reduce metabolic fuel availability [167]. In addition, iron deficiency affects reproductive function and bone metabolism [168] while several iron-dependent enzymes influence metabolic and immune responses [165]. Hepcidin response appears to be inversely related to EA, while an adequate EA might attenuate the inflammatory response to strenuous exercise [169]. Furthermore, decreased endogenous E2 is associated with higher levels of hepcidin [170].

### Summary Part B

Menstrual dysfunction alone is not a problem for sports performance, but the underlying altered endocrine function, characterized by sex hormone deficiencies and overall endocrine dysfunction, contributes to impairments in mechanisms that affect health and sports performance.

## LEA-Induced Endocrine Dysfunction Leads to Performance Decrements

While it is reasonable to infer that endocrine dysfunction caused by LEA interferes with training adaptations and performance measures, the research clearly linking endocrine dysfunction to blunted training adaptations or performance decrements is limited and relies, in large part, on self-reported menstrual status and a limited number of performance measures (Table 1). Nevertheless, it is imperative to understand that the impact of hormonal perturbations associated with LEA can be both vast and profound, affecting the homeostasis of various body systems that affect health, as well as training responses/adaptations and sports performance. Healthy training days are essential for long-term athlete development and ultimately sports performance. Therefore, it is important to recognize that hormonal dysfunction (concomitant with nutritional deficiencies/LEA) can affect training quality before menstrual dysfunction is identified. Importantly, adequate EA, energy stores, macronutrient availability, and intermediate metabolites are critical for maintaining quality training sessions with appropriate training volume, intensity, and recovery [171]. Thus, athletes may experience a reduction in training quality or recovery even after short-term LEA [172], although more severe consequences are likely to be experienced after long-term LEA.

At present, relatively little is known about the effects of LEA on maximal aerobic capacity and anaerobic thresholds, although performance decrements and impaired development in endurance performance have been observed [15, 16], which may be related to impaired metabolism and cardiovascular function. One possible mechanism to explain impaired exercise metabolism during LEA is decreased E2 [173]. Lower E2 levels are associated with lower levels of glycogen sparing and fat oxidation [174], while higher levels of E2 promote the availability and metabolism of free fatty acids as well as glucose availability and uptake into type I (oxidative) muscle fibers, although this may be attenuated by higher levels of P4. Exercising at higher intensities and producing force may be hindered during LEA because of reduced muscle glycogen [175] while endurance capacity may be impaired by a decreased ability to utilize fat. Sufficient muscle glycogen stores are necessary for exercise performance, and replenishing these stores is critical for recovery and sustained training [176, 177] where insulin facilitates the transport of glucose into muscle cells (at rest), a process that may be inhibited via decreased levels of E2 [178]. On a cellular level, mitochondrial biogenesis and function (metabolism and morphology) are also known to be influenced by E2 [179] while regulation of mitochondrial dynamics allows the cell to respond and adapt to cellular energy demands [180]. Thyroid hormones also stimulate mitochondrial biogenesis, energy metabolism, and energy transfer while influencing recovery. Mitochondria are essential for the generation of ATP via oxidative phosphorylation in response to energy depletion via AMP-activated protein kinase [181]. Exercise/training should enhance muscle metabolism, but reduced levels of E2 [28] and T3 resulting from LEA [182] could, in theory, blunt or block expected mitochondrial responses [183] and adaptations [19]. Mitochondrial oxidative functions and protein translation appear to be suppressed by LEA and appear to recover after refeeding [184].

Another mechanism affecting endurance performance could be impaired hematopoiesis, as evidenced for example, by lower erythrocyte and platelet counts, and increased white blood cell count in dieting fitness athletes [143]. Prolonged energy restriction and intense exercise training can also reduce iron stores, which, as discussed earlier, are important for oxygen delivery and transport, energy metabolism, cognition, and immune function [165, 185]. It is important to understand that the hormonal changes associated with LEA may be responsible for reduced blood flow related to impaired endothelial function, decreased fat oxidation related to mitochondrial dysfunction, and decreased hematopoiesis associated with impaired iron metabolism or decreased iron stores. We suggest that the synergistic effects of low E2, low T3, and low glycogen may impact mitochondrial remodeling processes, impairing aerobic metabolism and adaptations to endurance training in the longer term with impaired ATP production affecting force production in addition to the cellular repair required for recovery. The literature is currently lacking in studies addressing performance directly, but we postulate that the aforementioned hormonal perturbations and resulting health and functional challenges directly affect training quality, recovery, and performance.

LEA and nutritional deficiency appear to be strongly associated with impairments in muscle protein synthesis [131, 134, 186] and impaired neuromuscular function [19], which may result in blunted/decreased development in force production capabilities. Decreased blood glucose levels and hormonal disruptions in amenorrheic athletes have been associated with lower strength and lean mass of the lower extremities compared with eumenorrheic athletes [19]. A decrease in glycogen due to LEA may be problematic for muscle contraction (myosin cross-bridge interaction) owing to an impaired release of calcium from the sarcoplasmic reticulum [187]. Likewise, amenorrhea may affect metabolism during exercise recovery, possibly impairing the ability of amenorrheic athletes to, for example, optimally complete repeated bouts of exercise [183]. We might also hypothesize that LEA-induced low E2 has a negative influence on force production via central mechanisms [188]. Neuromuscular function and fatigability of the knee extensors change across the menstrual cycle, with greater intracortical inhibition and fatigue during the luteal phase and greater voluntary contraction when the E2 level is elevated [189]. Estradiol is known to alter neuronal excitability and may affect force production capacity via neurotransmitter receptors (direct) and ion channel-activated (indirect) mechanisms. An excitatory neuronal effect is associated with E2, whereas an inhibitory effect is associated with P4 [190]. Thus, in theory, a decrease in E2 related to LEA could reduce cortical excitability due to decreased action on sodium channels that results in attenuated recruitment of excitatory interneurons [191], which may also influence motor control and recruitment of motor units, although this has not been investigated in females with menstrual dysfunction versus those with eumenorrhea. Nevertheless, reduced neuromuscular function due to LEA and LEA-induced hormonal changes may impair mechanical efficiency, which could also increase the individual’s perception of loading. Importantly, even short-term and adaptable LEA may not be without consequences for recovery [172] and longer term adaptations. Differentiating between desired decreases (planned overreaching) and LEA-induced decreases in performance and recovery during training blocks may be difficult but important for long-term athlete development.

## Summary and Limitations

**Fig. 2:**
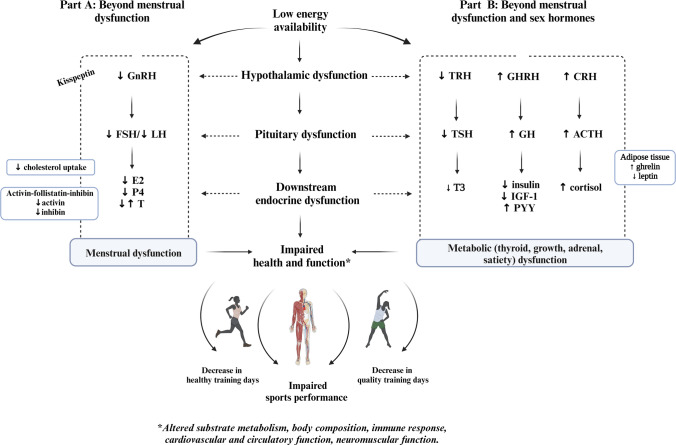
Summary of Part A: beyond menstrual dysfunction and Part B: beyond menstrual dysfunction and sex hormones. The endocrine system includes various points for physiological crosstalk and hormones often have pleiotropic effects (see reference [192]). Created with www.biorender.com. *ACTH* adrenocorticotropic hormone, *CRH* corticotropin-releasing hormone, *E2* estradiol, *FSH* follicle-stimulating hormone, *GH* growth hormone, *GHRH* growth hormone-releasing hormone, *IGF-1* insulin-like growth factor 1, *LH* luteinizing hormone, *P4* progesterone, *PYY* peptide YY, *T* testosterone, *T3* triiodothyronine, *TRH* thyrotropin-releasing hormone, *TSH* thyroid-stimulating hormone, ↑ increased, ↓ decreased

In female individuals, sex hormones are not only responsible for reproduction, but also play important roles in bone, muscle, and cardiovascular health and function. Menstrual dysfunction secondary to LEA is characterized by changes in hormonal profiles with the combined direct and indirect effects of E2 and P4 on an athlete’s ability to train and recover optimally [4, 14, 22]. Although menstrual dysfunction is indicative of suppression of sex hormones, the concomitant disturbances in other hormonal axes and their impact on athlete health and sports performance must be recognized. Indeed, the hormonal consequences of LEA appear to be controlled, in large part, by the hypothalamus, which connects the nervous system to the endocrine system via the pituitary gland. Low energy availability-induced changes in the levels of several pituitary hormones appear to have unfavorable downstream effects on structural characteristics (muscle protein turnover, adiposity, bone density), energetics (resting and exercise metabolism, mitochondrial function), and adaptation (strength, power, and endurance capacity) of the skeletal muscle and adipose tissue, with direct and indirect negative effects on sports performance (Fig. 2).

While it is understood that problematic LEA disrupts menstrual function, the evidence for dysfunction in other endocrine axes appears to be more scattered. In many cases, information regarding problematic LEA is drawn from studies including amenorrheic athletes or patients with anorexia nervosa. The endocrine system, in general, is regulated by several feedback loops that include various points for physiological crosstalk in which hormones often have pleiotropic effects. Given that hormones play a significant role in maintaining normal physiological function and supporting homeostasis of tissues and processes essential for health, it is reasonable to hypothesize, in line with REDs, that hormonal changes resulting from LEA may negatively affect training responses, adaptations, and performance. Because the temporal relationship between hormonal changes and physiological effects is variable, it is important to recognize that hormonal changes may induce physiological effects that are not always immediate, or even in the same time frame as the physiological responses or adaptations.

Generally, research examining the effects of LEA on health has been laboratory based with a “prescribed” EA that may not translate directly to the field or practical “free-living” situations [20, 69]. In addition, the long-term effects of LEA on performance, training adaptations, and recovery have often been investigated using a cross-sectional approach comparing athletes who already have menstrual dysfunction with naturally menstruating or eumenorrheic athletes. Much of this existing research also relies on self-reporting of menstrual status (i.e., “naturally menstruating” female individuals without hormonal level verification [28]). Finally, female individuals using HCs are not immune to the effects of LEA, although possible endocrine and or performance consequences specific to HC users have not yet been elucidated.

Although several ethical issues may prevent researchers from conducting long-term laboratory-based (especially long-term and severe) LEA studies in athletes, and it would be unethical not to intervene in free-living conditions if an athlete exhibits symptoms or behaviors indicating LEA, a schedule of regular hormonal and physical testing for groups of athletes could allow researchers to elucidate the time course of possible hormonal and performance changes occurring in athletes. Athletes should have access to a network of specialists when faced with REDs [193].

## Key Findings and Practical Applications

It is worth noting that the etiology behind menstrual dysfunction is not always LEA, but that menstrual dysfunction is indicative of marked hormonal changes that should be assessed by a physician. Additionally, menstrual dysfunction does not immediately translate into performance decrements, although the changes in hormonal profiles may ultimately be profound and detrimental to the health and performance of the athlete. Identification of REDs and hormonal dysfunction should be based on a comprehensive medical evaluation of symptoms (involving a multidisciplinary team), hormone testing, and exclusion of other medical problems.

### Key Findings and Practical Applications

The scientific evidence clearly linking endocrine dysfunction to decreased performance and blunted or decreased training adaptations is limited. We have described how LEA-induced changes in sex hormones that often manifest as menstrual dysfunction and concomitant hormonal dysfunction in other axes could result in several undesirable health outcomes including negative bone health, impaired metabolic activity, undesired outcomes for body composition, altered immune response and gut health, problematic cardiovascular outcomes, and iron deficiency that both directly and indirectly affect training and performance. While it is possible that short-term LEA will not markedly affect performance, it is important to investigate LEA-induced outcomes and their mechanisms in order to better understand the performance decrements associated with the Triad/REDs. As such, we suggest that mechanisms described in this article are influenced by altered endocrine function secondary to LEA and that these impair health and sports performance in female athletes. Based on the totality of the evidence, we suggest that researchers and practitioners:

- Explore the mechanisms by which endocrine dysfunction, including menstrual dysfunction, affects athlete performance, including the time course of performance decrements and changes in hormonal profiles.
- Recognize that present cross-sectional studies generally use only FHA as an indicator of prolonged LEA, although menstrual dysfunction such as oligomenorrhea or recurrent anovulation may indicate LEA.
- Consider the depth and breadth of LEA and the subsequent effects on hormonal homeostasis in free-living conditions (and consider the current literature) [194].
- Acknowledge that the negative effects of LEA are likely to begin before identifiable menstrual dysfunction, such as FHA. Perturbations in E2 and P4 occur even in less severe forms of menstrual dysfunction while other hormonal axes are also affected. This highlights the importance of going beyond monitoring menstrual bleeding alone and including methods to determine the more subtle menstrual dysfunction, such as monitoring ovulation and/or the P4 peak in the luteal phase [28].
- Understand that gynecological age may influence responses to LEA. Older female individuals and female individuals with greater gynecological age, i.e., years since onset of menarche, may be more adaptable to LEA than younger female individuals or female individuals of younger gynecological age [41].
- Monitor markers of menstrual function in female individuals not using HCs (including hormonal intrauterine devices). This may include menstrual bleeding along with an LH surge associated with ovulation (using an ovulation test [47, 48]), P4 peak in the luteal phase, and/or other frequent hormonal sampling [28].
- Consider the effects of LEA on HC users compared to non-users, as exogenous sex steroids may influence HPO axis function independently of other hormonal axes. Avoid including HC users in “mixed groups” with naturally menstruating/eumenorrheic or amenorrheic participants, as this may affect the interpretation of subsequent results.
- Monitor HC using athletes by assessing nutritional status proactively.
- Consider assessing surrogate markers of LEA, including but not limited to T3, testosterone, cortisol, IGF-1, and insulin, in addition to sex hormones, as well as lipid profiles, iron, gut health, and immune function in athletes with LEA or at risk of LEA.

## Conclusions

Suppression of sex hormones secondary to problematic LEA often manifests as menstrual dysfunction; however, concomitant hormonal dysfunction occurs in other endocrine axes. Taken together, this LEA-induced hormonal dysfunction underpins adverse mechanisms and outcomes that ultimately affect athlete health and impair training quality, thus likely negatively affecting performance. The influence of LEA-induced altered endocrine function on mechanisms of athlete health and components of sports performance requires further research.
